# Prenatal polycyclic aromatic hydrocarbon exposure and asthma at age 8-9 years in a multi-site longitudinal study

**DOI:** 10.21203/rs.3.rs-3129552/v1

**Published:** 2023-07-11

**Authors:** Allison R. Sherris, Christine T. Loftus, Adam A. Szpiro, Logan Dearborn, Marnie F. Hazlehurst, Kecia N. Carroll, Paul E. Moore, Margaret A. Adgent, Emily S. Barrett, Nicole R. Bush, Drew B. Day, Kurunthachalam Kannan, Kaja Z. LeWinn, Ruby H.N. Nguyen, Yu Ni, Anne M. Riederer, Morgan Robinson, Sheela Sathyanarayana, Qi Zhao, Catherine J. Karr

**Affiliations:** University of Washington; University of Washington; University of Washington; University of Washington; University of Washington; Icahn School of Medicine at Mount Sinai; Vanderbilt University Medical Center; Vanderbilt University Medical Center; Rutgers, The State University of New Jersey; University of California, San Francisco; Seattle Children’s Hospital; New York University; University of California, San Francisco; University of Minnesota; University of Washington; University of Washington; New York University; Seattle Children’s Hospital; University of Tennessee Health Science Center; University of Washington

**Keywords:** PAHs, asthma, children’s health

## Abstract

**Background and aim:**

Studies suggest prenatal exposure to polycyclic aromatic hydrocarbons (PAHs) may influence wheezing or asthma in preschool-aged children. However, the impact of prenatal PAH exposure on asthma and wheeze in middle childhood remain unclear. We investigated these associations in diverse participants from the ECHO PATHWAYS multi-cohort consortium.

**Methods:**

We included 1,081 birth parent-child dyads across five U.S. cities. Maternal urinary mono-hydroxylated PAH metabolite concentrations (OH-PAH) were measured during mid-pregnancy. Asthma at age 8–9 years and wheezing trajectory across childhood were characterized by caregiver reported asthma diagnosis and asthma/wheeze symptoms. We used logistic and multinomial regression to estimate odds ratios of asthma and childhood wheezing trajectories associated with five individual OH-PAHs, adjusting for urine specific gravity, various maternal and child characteristics, study site, prenatal and postnatal smoke exposure, and birth year and season in single metabolite and mutually adjusted models. We used multiplicative interaction terms to evaluate effect modification by child sex and explored OH-PAH mixture effects through Weighted Quantile Sum regression.

**Results:**

The prevalence of asthma in the study population was 10%. We found limited evidence of adverse associations between pregnancy OH-PAH concentrations and asthma or wheezing trajectories. We observed adverse associations between 1/9-hydroxyphenanthrene and asthma and persistent wheeze among girls, and evidence of inverse associations with asthma for 1-hydroxynathpthalene, which was stronger among boys, though tests for effect modification by child sex were not statistically.

**Conclusions:**

In a large, multi-site cohort, we did not find strong evidence of an association between prenatal exposure to PAHs and child asthma at age 8–9 years, though some adverse associations were observed among girls.

## 1. Introduction

Asthma is a leading chronic illness among children in the U.S., with prevalence and morbidity disproportionately affecting low-income children and those from racial-ethnic minoritized groups (Akinbami et al., 2014; Bryant-Stephens, 2009; Gold & Wright, 2005). Asthma presentation in children is heterogeneous, with phenotypes characterized by differences in wheezing symptoms throughout childhood, allergic sensitization, and asthma exacerbation and severity (Depner et al., 2014; Trivedi & Denton, 2019). Maternal environmental exposures during pregnancy are likely relevant to the etiology of asthma, though risk factors for asthma development and exacerbation differ among asthma phenotypes (Henderson et al., 2008; Salam et al., 2004; Sherriff et al., 2001; Taussig et al., 2003; Trivedi & Denton, 2019). Prenatal exposure to ambient air pollution, including particulate matter (PM), has been implicated as a risk factor for asthma in early life (Hehua et al., 2017). However, the specific components of air pollution that may drive adverse outcomes and their relationship with different asthma phenotypes are not well-understood.

Polycyclic aromatic hydrocarbons (PAHs) are component of ambient air pollution with established adverse impacts on child airway health (Kim et al., 2013). PAHs originate from the burning of organic materials including fossil fuels, biomass, tobacco products, and food (Baek et al., 1991). Some low-molecular weight PAHs, including naphthalene, also have industrial and commercial household sources including moth balls and deodorizers (Jia & Batterman, 2010). PAHs may be volatilized in the gas phase or bind to ambient fine particulate matter, resulting in exposure through the inhalation pathway. Exposure to PAHs may also occur through ingestion of grilled or charred food. Many low-molecular weight PAHs are metabolized to monohydroxylated metabolites (OH-PAHs), which are commonly detected in urine in the general population and among pregnant individuals (Woodruff et al., 2011). Prior research on the health effects of PAHs has largely focused on high molecular weight (HMW) PAHs and their carcinogenic properties (Armstrong et al., 2004; Boffetta et al., 1997; IARC Working Group on the Evaluation of Carcinogenic Risks to Humans, 2010; Korsh et al., 2015). However, recent studies suggest that exposure to PAHs may also affect airway development and function through disruption of endocrine and immune function and/or inflammation and oxidative stress (Kim et al., 2013; Wang et al., 2017; Zhu et al., 2021).

Prior research on the airway impacts of PAH exposure has predominantly focused on postnatal and occupational exposures (Jung et al., 2014g et al., 2020; Liu et al., 2016). Studies on prenatal exposure to PAHs and child airway outcomes have yielded mixed findings, with suggested associations between exposure to HMW PAHs and child asthma (Jung et al., 2011; Miller et al., 2004) and wheeze (Jedrychowski et al., 2010, 2014). The largest and most geographically diverse prior study, conducted among participants in the multi-site ECHO-PATHWAYS consortium, linked maternal urinary metabolites of pyrene and low molecular weight (LMW) PAHs (naphthalene, phenanthrene, and fluorene) to asthma among children aged 4–6 years, but associations were sex-dependent and observed only among females (Loftus et al., 2022). Existing research is characterized by differences in method of exposure assessment, prenatal exposure windows, outcome classification, and age of outcome assessment (Låg et al., 2020). In addition, most prior studies have focused on respiratory outcomes such as wheeze in early childhood, an age when asthma may be difficult to diagnose and objective measurements of airway obstruction and hyperreactivity are often not feasible (NHLBI, 2007).

This study expands on this literature to evaluate associations between prenatal PAH exposure and asthma among children in middle childhood (age 8–9 years), an age when persistent asthma can be identified and is more readily diagnosed compared to earlier years. We combine two cohorts of the ECHO-PATHWAYS Consortium across five U.S. cities, utilizing prenatal urinary OH-PAH concentrations in a diverse population to explore the role of individual OH-PAHs and metabolite mixtures. To interrogate whether maternal OH-PAH levels influence the development of particular asthma phenotypes, we leverage comprehensive longitudinal survey data in early and middle childhood to explore associations with wheezing trajectories (early, late, and persistent wheezing), atopic asthma, and asthma with recent exacerbation. Finally, we evaluate whether associations are modified by child sex.

## 2. Methods

### 2.1 Study population

This study population included pregnant individuals and their children followed from delivery (birth parent-child dyads) from two cohorts of the ECHO-PATHWAYS consortium: the Conditions Affecting Neurocognitive Development and Learning in Early Childhood (CANDLE) study and The Infant Development and the Environment Study (TIDES) (LeWinn et al., 2022). CANDLE is a socio-demographically diverse pregnancy cohort in in Shelby County (Memphis), Tennessee, originally established to identify early-life risk factors affecting child neurodevelopment. Participants were recruited during the second trimester of pregnancy from prenatal care clinics and the community in 2006–2011 (Sontag-Padilla et al., 2015). Inclusion criteria were: age 16–40 years, residence and planned delivery at an affiliated clinic in in Shelby County, low-risk singleton pregnancy, and English language proficiency at enrollment.

TIDES is a pregnancy cohort with four sites across the United States (San Francisco, CA; Minneapolis, MN; Rochester, NY; and Seattle, WA) and was originally established to evaluate the role of chemical exposures in early life reproductive development (Barrett et al., 2014). Inclusion criteria were: age of at least 18 years old, planning to deliver at an affiliated clinic, and having a low-risk pregnancy. In both CANDLE and TIDES, dyads were followed at regular intervals, including clinic visits and respiratory health questionnaires administered at age 4–6 years and age 8–9 years.

Inclusion criteria for the present analysis were: urinary OH-PAH and specific gravity (SG) measurements in the second trimester of pregnancy and airway outcome data at the age 8–9 year visit, including whether children had ever been diagnosed with asthma by a physician. We excluded participants with very preterm birth (gestational age less than 32 weeks) or evidence of prenatal smoking (based on maternal self-report and/or maternal urinary cotinine concentration above 200 ng/mL during pregnancy) due to competing risk factors and potentially distinct etiology of airway compromise in these populations. All ECHO-PATHWAYS research activities were approved by the University of Washington IRB and cohort study procedures were approved at each local institution.

### 2.2 Exposures

Urinary OH-PAH concentrations were measured in spot urine samples collected in mid-pregnancy, at a median gestational age of 21.6 weeks. Analytical procedures were performed at the Wadsworth Laboratory, New York State Department of Health and have been described in detail previously (Guo et al., 2013). In brief, liquid–liquid extraction was performed followed by identification and quantification of OH-PAHs with a ABSCIEX 5500 triple quadrupole mass spectrometer (Applied Biosystems; Foster City, CA, USA). Recoveries of analytes in Standard Reference Materials (SRM 3672, SRM 3673) ranged from 79 to 109%. Twelve metabolites of seven parent PAHs were quantified in urine samples: two metabolites of naphthalene (1-hydroxynaphthalene [1-NAP], 2-hydroxynaphthalene [2-NAP]), four metabolites of phenanthrene (2-hydroxyphenanthrene [2-PHEN], 3-hydroxyphenanthrene [3-PHEN], 4-hydroxyphenanthrene [4-PHEN], combined 1/9-hydroxyphenanthrene [1/9-PHEN]), 1-hydroxychrysene (1-CHRY), 6-hydroxychrysene (6-CHRY), 2/3/9-hydroxyfluorene (2/3/9-FLUO), 1-hydroxypyrene (1-PYR), 3-hydroxybenzo[c]phenanthrene (3-BCP), and 1-hydroxybenzo[a]anthracene (1-BAA). Limits of detection ranged from 0.003 ng/mL to 0.04 ng/mL and were generally higher in CANDLE relative to TIDES, with the exception of 1-NAP and 2/3/9-FLUO (Table S1). Samples below the limit of detection (LOD) were imputed as LOD/√2 for the primary analyses and for descriptive statistics. As a sensitivity analysis, we used an alternate multiple imputation approach described below. Urinary SG was determined using a handheld refractometer (Atago PAL-3 pocket refractometer) at the time of sample collection. For descriptive statistics and mixtures analysis, we accounted for urinary dilution by adjusting OH-PAH concentrations for SG using the Levine-Fahey equation (Boeniger et al., 1993). In regression models, we used raw, unadjusted OH-PAH concentrations with model adjustment for urinary SG.

This analysis included five metabolites detected in > 60% of the analytic population of both cohorts: 1-NAP 2-NAP 2-PHEN, 3-PHEN, and 1/9-PHEN (Table S2). Two metabolites, 2/3/9-FLUO and 1-PYR, were detected in > 60% of CANDLE participants (but not TIDES participants) and were additionally included in CANDLE-only models. One metabolite, 4-PHEN, was detected > 60% of TIDES participants (but not CANDLE participants) and was included in TIDES-only models.

### 2.3 Outcomes

The primary outcomes for this analysis were 1) current asthma at age 8–9 years and 2) wheezing trajectories in childhood, defined based on caregiver responses to standard International Study of Asthma and Allergies in Childhood (ISAAC) questionnaire (Asher et al., 1995) at the age 4–6, and 8–9 visit. *Current asthma* at age 8–9 was defined as caregiver-reported physician diagnosis of asthma and either current asthma medication use or recent (in the 12 months prior to the clinic visit) wheezing or whistling in the chest. Wheezing trajectories were derived from ISAAC questionnaire responses from the age 4–6 and 8–9 year visits: *never wheezing* (no history of wheeze at age 4–6 or age 8–9 years), *early wheezing* (affirmative history of wheeze at age 4–6 but no recent wheeze at age 8–9 years), *late wheezing* (no history of wheeze at age 4–6 but affirmative history of wheeze at age 8–9 years); and *persistent wheezing* (history of wheeze at age 4–6 and recent wheeze at age 8–9 years). These categories reflect clinical differences in wheezing and asthma phenotypes observed throughout childhood (Depner et al., 2014; Martinez et al., 1995; Trivedi & Denton, 2019).

Two secondary outcomes were evaluated: *Current asthma with recent exacerbation* required current asthma at age 8–9 (as defined above) and either oral/injected steroid use in the past 12 months or hospitalization due to asthma or wheezing in the past 12 months. *Combined asthma with atopic disease* was defined as current asthma with history of allergic rhinitis / hay fever or history of eczema.

### 2.4 Statistical analysis

We used logistic regression to estimate the odds ratios and 95% confidence intervals for asthma associated with prenatal exposure to individual OH-PAHs. To evaluate associations between individual OH-PAHs and wheezing trajectories, we used multinomial regression with “never wheezing” as the reference category. Unadjusted OH-PAH concentrations were log-transformed with base-2, and we report outcomes associated with a doubling in OH-PAH levels.

We performed separate logistic regression models for each individual urinary OH-PAH concentration. Covariates were selected *a priori* and were identified as confounders directly or indirectly associated with prenatal PAH exposure and child airway outcomes, or as precision variables that were associated with airway outcomes alone and may improve model estimates. Models were adjusted for potential confounders in a staged approach. Minimally-adjusted models included urinary SG and child age as continuous variables and child sex and study site as categorical variables. Fully-adjusted models were additionally adjusted for SG, cohort, maternal age (continuous), education at enrollment (less than high school, high school completion, graduated college/technical school, or any graduate school), self-identified race (collapsed into categories of Black/African American, White, or Other due to small sample size in some subgroups), and ethnicity (Hispanic/not Hispanic), season of birth (warm or cold), inflation-adjusted household income interacted with household size, neighborhood deprivation index at the residential address during pregnancy (continuous), prenatal environmental tobacco smoke (ETS) exposure (continuous maternal urinary cotinine), reported postnatal ETS exposure (binary), maternal history of asthma (binary), firstborn status (binary), and the interaction between SG and cohort.

We evaluated associations between individual OH-PAHs and secondary asthma phenotypes (current asthma with recent exacerbation and combined asthma plus atopic disease) using logistic regression adjusting for covariates in the staged approach described above. We explored effect modification by child sex using multiplicative interaction terms between OH-PAH concentrations and child sex.

As a secondary aim exploring associations between OH-PAH mixtures and child asthma and wheeze, we used logistic weighted quantile sum (WQS) regression (Carrico et al., 2014). In brief, WQS regression is a two-stage process; first, a weighted index of quantized mixture components is estimated to reflect components most strongly associated with outcome in either a positive or negative direction. Next, the association between the weighted index and the outcome is estimated to reflect the “overall mixture effect”. We implemented WQS models using decile transformation and N = 1000 bootstraps while using the same data for model training and validation to identify PAH index weights and mixture effects in both the positive and negative direction of association with the primary outcomes (asthma and wheezing trajectories). Models were adjusted for the covariates described in the full-adjusted model for Aim 1. We elected *a priori* to use a novel permutation test to evaluate to control Type I error in the event of statistically significant associations between the WQS index and airway outcomes (Day et al., 2022; Loftus et al., 2022),

As a sensitivity analysis, we evaluated associations between mutually-adjusted OH-PAHs and primary and secondary airway outcomes (rather than separate models for each metabolite). Additional sensitivity analyses were performed for the primary outcome of asthma at age 8–9, including: 1) cohort-specific models and models excluding one TIDES site sequentially to account for potential heterogeneity in the population and site-specific differences confounding; 2) models using SG-adjusted urinary OH-PAH values in addition to adjustment for SG; 4) imputation of OH-PAH measurements below the detection limit with central likelihood multiple imputation (CLMI) (Boss et al., 2019), a method for imputation in settings where limits of detection vary by analytical batch, instead of LOD/√2; 5) additional adjustment for birth weight and gestational age as potential mediators and birth year to account for secular trends; 7) multiple imputation with chained equations (MICE) for missing covariate data; and 8) inverse-probability-of-selection weighting to correct potential selection bias from differential loss-to-follow-up.. All analyses were performed using R version 4.2.2 (R Core Team, 2022) and the packages mice (Buuren et al., 2023), lodi (Boss & Rix, 2020), and gWQS (Renzetti et al., 2021).

## 3. Results

### 3.1 Study population characteristics

There were N = 2,403 birth parent-child dyads in the CANDLE and TIDES cohorts, including N = 1,662 with OH-PAH and SG measurements during mid-pregnancy. Of these, N = 1,222 attended follow-up visits at age 8–9 years and provided answers to ISAAC questionnaires. We excluded N = 123 participants with evidence of maternal smoking during pregnancy and N = 18 children with gestational age < 32 weeks. After exclusion, the study population comprised 1,081 participants representing 698 CANDLE dyads and 383 TIDES dyads (Seattle, WA: n = 93; San Francisco, CA: n = 79; Minneapolis, MN: n = 118; Rochester, NY: n = 93). The demographic composition of CANDLE and TIDES participants excluded from the study population was similar to those included, though the study population had higher income on average than those excluded (Table S3).

Children in the study population were 52% male and ranged in age from 8–9 years, with over half of outcome assessments performed at 8 years of age (Table 1). The study population was demographically diverse, with 44% of birth parents identifying as Black or African American and 47% as White. There were notable differences in the sociodemographic characteristics of participants from the CANDLE and TIDES cohorts. Most CANDLE birth parents were Black or African American (62%) and 46% had a college/technical degree or higher. In contrast, the majority of TIDES birth parents were White (74%) and 83% graduated college. The median adjusted household income in TIDES was greater than three times that of CANDLE households.

### 3.2 Pregnancy OH-PAH exposure

Rates of OH-PAH detection (Table S2) and concentration distributions (Table S1) differed among metabolites and by cohort. Detection rates ranged from 85% (1/9-PHEN) to 100% (2-NAP) in the overall study population. In general, median SG-adjusted OH-PAH concentrations were higher in CANDLE than TIDES. Median concentrations of naphthalene metabolites (1-NAP and 2-NAP) were higher than those of phenanthrene metabolites (2-PHEN, 3-PHEN, and 1/9-PHEN). The strongest pairwise correlations were observed between phenanthrene metabolites, with Pearson correlation coefficients ranging from 0.55–0.75 (Table S4). Correlation between other metabolites was moderate, with correlation coefficients ranging from 0.34–0.45.

We observed difference in OH-PAH exposure in the study population by cohort and sociodemographic characteristics. CANDLE participants were overrepresented among those in the highest tertile of urinary OH-PAH concentrations, relative to those in the lowest tertile (Table S5). Participants in the highest tertile of OH-PAH exposure were more likely to be Black or African American, were less likely to have graduated college at enrollment, and had lower mean household income relative to those in the lowest tertile.

### 3.3 Child airway outcomes

The prevalence of current asthma at age 8–9 years in the study population was 10%, representing 11.6% of CANDLE children and 7% of TIDES children (Table 2). About half of study participants were categorized as “never wheezing”; among children with a history of wheeze, most were in the “early wheezing” category, followed by “late” and “persistent wheezing”. Secondary asthma outcomes were less common in the study population, including asthma with recent exacerbation (5.0%) and asthma with atopic disease (6.8%).

### 3.4 Analytic results

**Figure 1:**
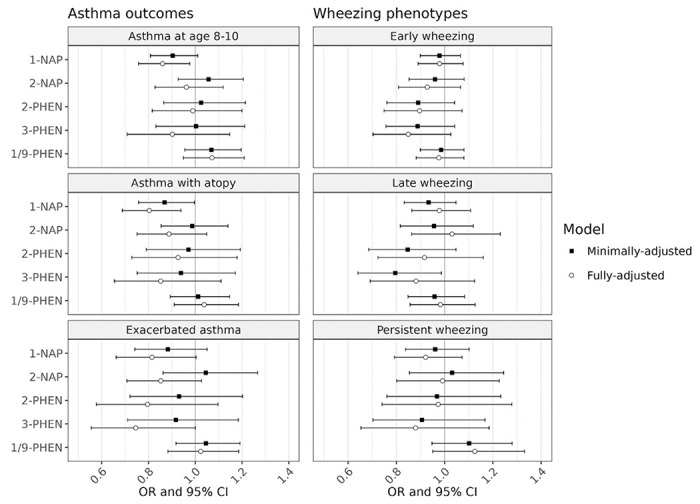
Associations between prenatal OH-PAH metabolites and asthma phenotypes at age 8-9 and wheezing phenotypes in childhood. Adjusted odds ratios (OR) and 95 % confidence intervals (CI) associated with a twofold increase in individual OH-PAH metabolite were determined by logistic regression (asthma outcomes) and multinomial regression (wheezing trajectories). Logistic regression was used to determine associations for asthma phenotypes, while multinomial regression was used for wheezing phenotypes. Minimally adjusted models were adjusted for child age at assessment, child sex, study site, and urinary specific gravity. Fully adjusted models were additionally adjusted additionally adjusted for the interaction between SG and cohort, maternal age, education at enrollment, race, and ethnicity; season of birth; inflation-adjusted household income interacted with household size, neighborhood deprivation index, prenatal smoke exposure, postnatal smoke exposure, maternal history of asthma, and firstborn status. Abbreviations: 1-NAP – 1-hydroxynaphthalene; 2-NAP – 2-hydroxynaphthalene; 2-PHEN – 2-hydroxyphenanthrene; 3-PHEN – 3-hydroxyphenanthrene; 1/9-PHEN – combined 1- and 9-hydroxyphenanthrene.

The association between individual OH-PAHs and all primary and secondary outcomes were null in minimally-adjusted models (Figure 1). In fully-adjusted models, there was a statistically significant inverse association between 1-NAP concentrations and asthma, with an odds ratio [OR] of 0.86 associated with a doubling in 1-NAP concentrations (95% confidence interval [CI]: 0.76, 0.98). We also observed inverse associations between 1-NAP and the secondary outcomes of asthma with recent exacerbation (OR = 0.76, 95% CI: 0.61, 0.95) and asthma with atopy (OR = 0.80, 95% CI: 0.69, 0.94).

**Figure 2:**
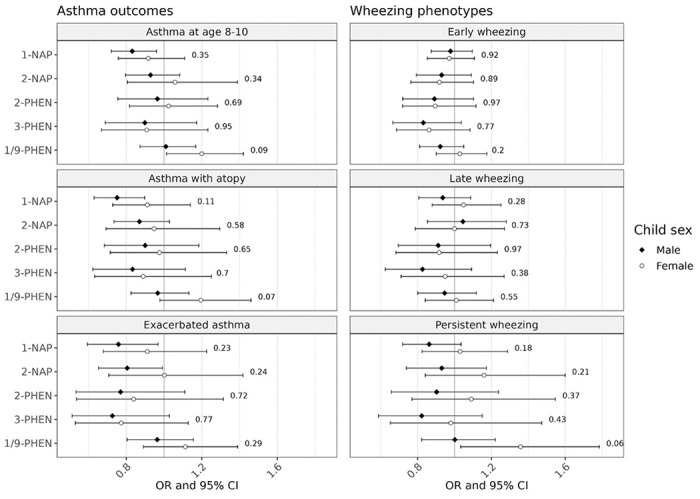
Effect modification by child sex in the association between airway outcomes and OH-PAHs, represented by adjusted odds ratios (OR) and 95% confidence intervals (CI) associated with twofold increase in individual OH-PAH. Strata-specific results were derived from interaction models; p-values are for the interaction term between child sex and OH-PAH concentrations. Models were adjusted for child age and sex; study site; the interaction between urinary SG and cohort; maternal age, education at enrollment, race, and ethnicity; season of birth; inflation-adjusted household income interacted with household size, neighborhood deprivation index, prenatal smoke exposure, postnatal smoke exposure, maternal history of asthma, and firstborn status. Abbreviations: 1-NAP – 1-hydroxynaphthalene; 2-NAP – 2-hydroxynaphthalene; 2-PHEN – 2-hydroxyphenanthrene; 3-PHEN – 3-hydroxyphenanthrene; 1/9-PHEN – combined 1- and 9-hydroxyphenanthrene.

In effect modification analyses, sex-specific differences were minor and not in a consistent direction for the outcomes of early wheezing and late wheezing (Figure 2). For asthma, a suggestive inverse association with 1-NAP among boys was attenuated among girls, though the p-value for the interaction term was not statistically significant (p_interaction_ = 0.35). We observed some adverse associations among girls only, including between 1/9-PFIEN and asthma (OR_female_ = 1.20, 95% CI: 1.01, 1.42; p_interaction_ = 0.09) and between 1/9-PHEN and persistent wheeze (OR_female_ = 1.36, 95% CI: 1.03, 1.78; p_interaction_ = 0.06), with borderline evidence for effect modification.

In WQS models, associations between the overall OH-PAH mixture (WQS index) and asthma were null in both the positive and negative direction (Table 3). There was a non-significant positive (adverse) association between a decile increase in the WQS index and child asthma in minimally-adjusted models (OR = 1.06, 95% CI: 0.96, 1.18) that was attenuated in fully-adjusted models (OR = 1.02, 95% CI: 0.91, 1.13), for which 1/9-PHEN dominated the WQS index mixture (weight = 0.56) (Table 3, Table S6). In WQS models describing negative (inverse) associations between the WQS index and asthma, there was a borderline inverse association in minimally-adjusted models that was attenuated in fully adjusted models, for which 1-NAP dominated the WQS index mixture (weight = 0.57).

In models mutually-adjusted for all OH-PAHs, associations between 1/9-PHEN and all primary and secondary outcomes were elevated relative to separate metabolite-specific models (Table S7). For example, we observed statistically significant associations between mutually-adjusted 1/9-PHEN and asthma (OR = 1.20, 95% CI: 1.01, 1.44) and persistent wheezing (OR = 1.32, 95% CI: 1.04, 1.67). Associations between 1/9-PHEN and asthma with recent exacerbation (OR = 1.21, 95% CI: 0.97, 1.51) and asthma with atopy (OR = 1.19, 95% CI: 0.98, 1.46) were also elevated in mutually-adjusted models.

We observed differences in cohort-specific sensitivity analyses of OH-PAHs and child asthma, with adverse associations (OR>1) for all OH-PAHs except 1-NAP in TIDES and protective associations (OR<1) for all metabolites except 1/9-PHEN in CANDLE (Table S8). The association between 1/9-PHEN and asthma was statistically significant in TIDES (OR = 1.49, 95% CI: 1.05, 2.21) but not CANDLE (OR = 1.01, 95% CI: 0.88, 1.15). The association between 4-PHEN and asthma–evaluated only among TIDES participants due to the low detection rate of 4-PHEN in CANDLE–was also found to be elevated (OR = 1.75, 95% CI: 1.07, 2.86). The results were robust to exclusion of individual TIDES sites (Table S9).

When values below the detection limit were imputed with CLMI, the negative association between 1-NAP was attenuated and no longer statistically significant; all other estimates were largely unchanged (Table S10). Other sensitivity analyses yielded results similar to those of primary models, including use of SG-adjusted OH-PAH concentrations instead of unadjusted metabolite concentrations; additional adjustment by birth weight, gestational age, and birth year; imputation of missing covariate data with MICE; and inverse probability of censoring weighting (Table S10).

## 4. Discussion

We evaluated associations between prenatal exposure to select PAH metabolites and childhood airway outcomes in a diverse, multi-site cohort with comprehensive covariate characterization. We did not observe strong evidence for associations between concentrations of urinary OH-PAH metabolites–individually or in mixtures–and child asthma at age 8-9 or wheezing trajectories in childhood. Counter to our hypothesis that PAH exposure has adverse impacts on airway health, urinary concentrations of 1-NAP were inversely associated with asthma in fully-adjusted models and most sensitivity analyses. This seemingly protective association was attenuated among girls, though evidence for effect modification was lacking. Concentrations of 1/9-PHEN were positively associated with asthma and persistent wheezing in several sensitivity analyses and among girls, with borderline evidence for effect modification.

Several prior studies have evaluated the association between maternal PAHs and child airway outcomes, though most have focused on early childhood outcomes based on reported respiratory signs and symptoms. Using a similar study design, Loftus et al (2022) explored links between maternal OH-PAHs and asthma and wheeze in early childhood (age 4-6 years) in the CANDLE and TIDES cohorts. The authors identified null associations for most exposure-outcome associations, though with borderline evidence of an adverse effect of 1-NAP and 1/9-PHEN on current asthma at age 4-6 years. Notably, evidence of effect modification by child sex was strong, with elevated associations between OH-PAHs (except for 1-NAP) and current asthma among girls only; interaction p-values were statistically significant for metabolites including 1/9-PHEN, 3-PHEN, 2-PHEN, and 2-NAP.

Other studies of prenatal exposure to PAHs and child airway outcomes have largely focused on HMW parent PAHs, yielding mixed results. Differences in specific analytes measured, study design, timing of exposure assessment, age of outcome assessment, and/or case definition among prior studies complicate comparison with the present analysis. Furthermore, these studies have generally relied on small sample sizes in relatively homogenous populations, derived from three pediatric cohorts. In a birth cohort of 339 mother-infant pairs in Krakow, Poland, researchers measured personal exposure to HMW PAHs including benzo(a)anthracene, benzo(b)fluoranthene, benzo(k)fluoranthene, benzo(g,h,i)perylene, benzo(a)pyrene, chrysene/iso-chrysene, dibenzo(a,h)anthracene, indeno(1,2,3-c,d)pyrene, and pyrene over 48 hours during the second trimester of pregnancy (Jedrychowski et al., 2005, 2014, 2015). Increased maternal PAH exposure was associated with increased number and duration of respiratory symptoms (including ear infection, cough, barking cough, wheezing without cold, and sore throat) during the child’s first year of life (Jedrychowski et al., 2005) and with increased incidence risk ratio of wheeze during the first four years of life (Jedrychowski et al., 2014). Prenatal exposure to PAHs was also assessed by DNA adducts of benzo(a)pyrene in umbilical cord blood in the same cohort; elevated benzo(a)pyrene DNA adducts were associated with increased risk of wheezing during the first two years of life, but not with wheezing events during the third and fourth year of life. (Jedrychowski et al., 2010).

In the Columbia Center for Children’s Environmental Health cohort, personal exposure to pyrene and 8 HMW PAHs (benz(a)anthracence, benzo(b)fluoranthene, benzo(k)fluoranthene, benzo(g,h,i)perylene, benzo(a)pyrene, chrysene/isochrysene, dibenz(a,h)anthracene, and indeno(1,2,3)pyrene) was measured over 48-hours during the third trimester of pregnancy among a sample of 303 Dominican and African American women in New York City and airway outcomes were measured throughout infancy and early childhood (Jung et al., 2014; Miller et al., 2004; Rosa et al., 2011). The researchers found prenatal PAH exposure in combination with environmental tobacco smoke exposure was associated with increased risk of cough and wheeze at 12 months and probable asthma at age 24 months, with significant interaction between PAH and ETS exposure (Miller et al., 2004). This interaction persisted at age 5-6, with associations between prenatal exposure and asthma observed only among children with exposure to ETS (Rosa et al., 2011). Higher levels of prenatal and postnatal pyrene exposure were also associated with increased asthma and wheeze among non-atopic children (but not children with allergic sensitization) at age 5–6 years (Jung et al., 2014).

Most prior studies have evaluated associations between prenatal PAH exposure and airway outcomes in early childhood. Our analysis additionally considers asthma and wheezing trajectories in middle childhood that may represent distinct etiologies or clinical presentations. We evaluated three wheezing phenotypes identified by The Tucson Children’s Respiratory Study (TCRS) based on clinical observations throughout childhood: early, late, and persistent wheeze (Martinez et al., 1995). Similar phenotypes have also been identified through data-driven analyses of wheezing and asthma symptoms in other cohorts (Henderson et al., 2008; Savenije et al., 2011). Children with these phenotypes have been found to have differences in allergic sensitization, lung function, genetic factors, and risk factors for asthma development (Trivedi & Denton, 2019). For example, early wheeze that resolved by middle childhood is generally associated with only mild lung function impairments that do not usually require medication use (Depner et al., 2014). Late-onset wheezing has been linked to high likelihood of allergic sensitization (Depner et al., 2014; Henderson et al., 2008). Children with both late-onset and persistent wheezing have been found to have decrements in lung function and airway responsiveness, while persistent wheezing is generally associated with the highest rate of asthma diagnosis (Depner et al., 2014; Henderson et al., 2008; Savenije et al., 2011).

Our primary analysis did not identify associations between maternal OH-PAHs and any wheezing phenotype, though 1/9-PHEN was significantly associated with persistent wheezing (but not other wheezing trajectories) in models mutually adjusted for other OH-PAHs and among girls only in effect modification analyses. Thus, we observed similar trends in effect estimates and modification by child sex for the outcomes of persistent wheezing and diagnosed child asthma at age 8-9 years, potentially suggesting a similar underlying mechanism and/or overlap in affected populations due to high rates of asthma diagnosis among children with persistent wheeze. While no prior studies have evaluated associations between prenatal PAH exposure and wheezing trajectories, a recent meta-analysis identified associations between postnatal traffic-related air pollution and transient and persistent asthma/wheezing, but not late-onset asthma/wheezing (Lau et al., 2018). Studies have also linked prenatal exposure to CO, NO_x_, and PM_2.5_ to persistent asthma/wheeze specifically (Pennington et al., 2018).

Our analysis of maternal OH-PAHs and secondary asthma phenotypes in children (asthma with recent exacerbation and asthma with atopy) did not yield strong associations. Findings from the Columbia Center for Children’s Environmental Health birth cohort suggested that maternal personal pyrene measurements during pregnancy were associated with child asthma and wheeze at 5-6 years only among non-atopic children (Jung et al., 2011, 2014; Jung, Yan, et al., 2012), and that prenatal PAH exposure was not associated with the development of seroatopy at age 5-6 years (Rosa et al., 2011). While few studies have evaluated prenatal PAH exposure in association with asthma exacerbation, Jung et al. (2011) identified associations between high prenatal pyrene levels and emergency room visits for asthma before age 5-6. Further research is needed to characterize the role of prenatal PAH exposure in the development of seroatopy, atopic vs nonatopic asthma, and asthma severity in childhood.

The predominantly null results in our study may indicate that maternal OH-PAH levels in our study population are not associated with child asthma and wheezing trajectories at age 8-9. The OH-PAH concentrations measured in our study were similar to those identified by Cathey et al. (2018) among pregnant individuals in Boston (N = 200) and Puerto Rico (N = 50) with the exception of 2-NAP, for which geometric mean concentrations were higher in participants from Puerto Rico relative to those in Boston and our study population. Associations between OH-PAH levels and airway outcomes may be more modest than our study was powered to detect, particularly for asthma phenotypes with low prevalence in the study population, or may be limited to OH-PAH concentrations higher than those measured in the study population. We did observe an inverse (protective) association between 1-NAP and asthma, which is not easily explained. There may be plausible mechanisms by which prenatal PAH exposure could prevent or reduce airway symptoms (Rosa et al., 2011), for example by inhibiting B cell growth (Mann et al., 2001) or inducing pre-B cell apoptosis (Allan et al., 2006), which could be hypothesized to downregulate IgE production and thereby lead to a decrease in respiratory symptoms related to atopic asthma. However, most prior research has focused on the mechanisms by which prenatal PAH exposure may adversely affect childhood airway outcomes. While these mechanisms are not fully understood, adverse associations have been hypothesized to derive from oxidative stress and inflammation induced by PAH exposure. For example, PAH exposure from diesel exhaust was shown to generate reactive oxygen species in macrophages and epithelial cells that activated inflammatory signaling pathways, leading to upregulation of genes involved in regulating immune response (Li et al., 2003). Both LMW and HMW PAHs are also lipid-soluble and transferred across the placenta, and inflammatory responses and oxidative stress induced by PAH exposure is also hypothesized to adversely affect fetal development (Autrup et al., 1995; Perera et al., 1999). Gestational exposure to PAHs may also trigger transcriptomic and epigenetic changes (Herbstman et al., 2012). For example, a recent study found that maternal urinary OH-PAH metabolites were associated with changes in the placental transcriptome (Paquette et al., 2023). In particular, phenanthrene metabolites showed strong associations with expression of TRIP13 and genes related to vitamin absorption/digestion, consistent with our finding of stronger adverse associations for phenanthrene metabolite 1/9-PHEN relative to other OH-PAHss.

We found some evidence for effect modification by child sex, with statistically significant associations between 1/9-PHEN and the outcomes of age 8-9 asthma and persistent wheeze among girls only. However, the interaction p-values for these associations reached only borderline statistical significance; in contrast, Loftus et al. (2022), identified strong evidence for effect modification by sex in the association between maternal OH-PAH levels and asthma and wheeze at age 4-6 in a similar study population. Few other studies have evaluated effect modification by child sex in the relationship between PAH exposure and airway outcomes. Liu et al. (2016) identified some elevated associations between urinary 2-PHEN and asthma at age 13-19 among boys and between 4-PHEN and asthma at age 13-19 among girls, though most exposure-outcome associations were not significantly different between sexes and no formal tests of interaction were conducted. Majewska et al. (2018) found that lung function growth trajectories were lower among girls exposed prenatally to HMW PAHs, but did not differ by exposure among boys, though the interaction between child sex and exposure was not statistically significant. A recent study of maternal OH-PAHs and placental gene expression may offer a potential mechanism to explain a stronger adverse effect on airway development and function in females; relative to males, placentas from female births had over 60% more genes for which expression was associated with OH-PAH concentrations, including genes related to vitamin absorption (Paquette et al., 2023). More research is needed to clarify potential effect modification by child sex and the role of specific parent compounds and metabolites.

Our findings were somewhat sensitive to model specification and certain stratified analyses. The associations between 1/9-PHEN and multiple outcomes (asthma, persistent wheeze, asthma with recent exacerbation, and asthma with atopy) were elevated in models mutually adjusted for all OH-PAHs, relative to individual metabolite models. Mutually-adjusted models have been used to control for confounding by co-occurring OH-PAHs, which showed moderate to high correlation between metabolite concentrations (rho = 0.34-0.75), but can also amplify bias an lead to inflation of effect estimates and variance (Chiu et al., 2018; Weisskopf et al., 2018). In cohort-specific analyses, the associations between all OH-PAHs except 1-NAP and current asthma were elevated in TIDES relative to CANDLE. However, cohort-specific estimates of associations were also imprecise with overlapping confidence intervals. Thus, the differences in estimates by cohort may be driven by random noise, particularly given the small sample size of TIDES participants relative to CANDLE.

Our study has several notable strengths. As described above, the use of airway data from multiple time points also enabled us to evaluate wheezing trajectories that may provide insight into wheeze phenotypes with distinct environmental risk factors and etiologies. In addition to longitudinal outcome assessment, we also leverage data on reported asthma diagnosis in middle childhood, when asthma persistence can be more easily identified and diagnosed relative to early childhood. Our analysis used prospective data from two cohorts across five cities, representing participants with diverse demographic and socioeconomic backgrounds. These cohorts also collected comprehensive data on covariates and precision variables, enabling a high degree of statistical adjustment for potential confounders.

Our study also has various limitations. First, our estimates of PAH exposure are derived from only one spot urine sample collected during mid-pregnancy. Urinary OH-PAHs represent short-term exposure, with half-lives in the range of 2.5 – 12 hours (Brzeźnicki et al., 1997; Li et al., 2012). Prior studies of urinary OH-PAH concentrations across pregnancy have identified intraclass correlation coefficients (ICCs) that vary widely between OH-PAH analytes and among sub-populations, ranging from 0.04 to 0.73 (Cathey et al., 2018; Dobraca et al., 2018; Gaylord et al., 2022; Zhu et al., 2021). Measurement from a single time point may not represent pregnancy average exposures, and noise introduced by short-term variations in exposure may contribute to null findings and obscure associations with adverse airway outcomes. Our exposures of interest also included only metabolites of LMW PAHs, namely naphthalene and phenanthrene, thus we did not capture the full range of PAHs potentially influencing asthma outcomes. HMW PAHs are predominantly excreted in feces, rather than urine (Agency for Toxic Substances and Disease Registry, 1995). Urinary metabolites of three HMW PAHs, including benz[a]anthracene, benzo[c]phenanthrene, and chrysene, were measured in our study but only detected in a small percentage of samples and were not included in the analysis. In addition, 1-NAP is a metabolite of both naphthalene and the carbamate pesticide carbaryl (Meeker et al., 2007) and so we cannot distinguish associations with either parent compound for this metabolite. We further cannot rule out the potential for residual confounding due to imprecisely measured or unmeasured covariates, including postnatal PAH exposure.

Both asthma and wheezing outcomes relied on maternal report of recent symptoms of airway obstruction and diagnoses and may be affected by imprecise recall. Misclassification of both outcomes would most likely be nondifferential with respect to exposure, potentially contributing to null findings. Longitudinal follow-up of children at more time points (e.g., annually) would result in more consistent and accurate measures of wheezing phenotypes. Nonetheless, the use of data from two time points in middle- and late-childhood to classify wheezing phenotypes has precedent in the literature (Abellan et al., 2022; Jung, Hsu, et al., 2012; Martinez et al., 1995) in situations where more frequent outcome assessment were not possible. Furthermore, access to reported asthma diagnosis in middle childhood is a strength of the study; prior research on PAH exposure and pediatric airway outcomes has largely focused on outcomes in early childhood, when asthma diagnosis is difficult and objective measurements of airway obstruction and hyperreactivity are often not feasible. Finally, clinical histories derived from ISAAC questionnaire data are widely used to classify asthma outcomes in epidemiological studies and clinical settings (NHLBI, 2007).

In conclusion, in this large, multi-city study of maternal urinary OH-PAHs and airway outcomes in middle childhood, we did not identify strong evidence for associations between OH-PAHs and child airway outcomes. We identified borderline evidence for effect modification by child sex, with adverse associations between 1/9-PHEN and asthma and persistent wheezing among girls. Future research into the potential sex-specific impacts of prenatal PAH exposure on airway health throughout childhood are merited. Replication studies, particularly those with repeated exposure assessment throughout pregnancy, would improve scientific understanding of these linkages and the role of specific PAH parent compounds and metabolites.

## Figures and Tables

**Table 1:** Characteristics of the study population by cohort and overall.

	CANDLE (N = 698)	TIDES (N = 383)	Overall (N = 1081)
**Child sex**			
Female	355 (50.9%)	204 (53.3%)	559 (51.7%)
Male	343 (49.1%)	179 (46.7%)	522 (48.3%)
**Child age at clinic visit (years)**			
8	394 (56.4%)	211 (55.1%)	604 (55.9%)
9	230 (33.0%)	146 (38.1%)	376 (34.8%)
10	74 (10.6%)	26 (6.8%)	100 (9.3%)
**First born**			
Yes	276 (39.5%)	200 (52.2%)	476 (44.0%)
No	422 (60.5%)	174 (45.4%)	596 (55.1%)
Missing	0 (0%)	9 (2.3%)	9 (0.8%)
**Postnatal second-hand smoke exposure**			
Yes	163 (23.4%)	7 (1.8%)	170 (15.7%)
No	518 (74.2%)	347 (90.6%)	865 (80.0%)
Missing	17 (2.4%)	29 (7.6%)	46 (4.3%)
**Maternal education at enrollment**			
Less than high school	58 (8.3%)	18 (4.7%)	76 (7.0%)
High school completion	311 (44.6%)	44 (11.5%)	355 (32.8%)
Graduated college or technical school	233 (33.4%)	132 (34.5%)	365 (33.8%)
Some or more graduate school	95 (13.6%)	186 (48.6%)	281 (26.0%)
Missing	1 (0.1%)	3 (0.8%)	4 (0.4%)
**Maternal race**			
Black or African American	433 (62.0%)	38 (9.9%)	471 (43.6%)
Multiple races	37 (5.3%)	10 (2.6%)	47 (4.3%)
White	219 (31.4%)	285 (74.4%)	504 (46.6%)
Other	9 (1.3%)	46 (12.0%)	55 (5.1%)
Missing	0 (0%)	4 (1.0%)	4 (0.4%)
**Maternal ethnicity**			
Hispanic or Latino	11 (1.6%)	24 (6.3%)	35 (3.2%)
Not Hispanic or Latino	687 (98.4%)	355 (92.7%)	1042 (96.4%)
Missing	0 (0%)	4 (1.0%)	4 (0.4%)
**Maternal history of asthma**			
Yes	118 (16.9%)	63 (16.4%)	181 (16.7%)
No	558 (79.9%)	310 (80.9%)	868 (80.3%)
Missing	22 (3.2%)	10 (2.6%)	32 (3.0%)
**Maternal urinary cotinine (ng/mL)**			
Median [Min, Max]	0.221 [0.00141, 197]	0.00919 [0.00919, 159]	0.0937 [0.00141, 197]
Missing	4 (0.6%)	0 (0%)	4 (0.4%)
**Adjusted household income (USD)**			
Mean (SD)	41,000 (28,400)	112,000 (61,200)	66,000 (54,700)
Median [Min, Max]	31,000 [2,490, 83,400]	111,000 [6,160, 213,000]	51,600 [2,490, 213,000]
Missing	40 (5.7%)	22 (5.7%)	62 (5.7%)

**Table 2:** Prevalence of airway outcomes in the study population by cohort and overall.

	CANDLE (N = 698)	TIDES (N = 383)	Overall (N = 1081)
**Asthma (8–9 y)**			
Yes	81 (11.6%)	27 (7.0%)	108 (10.0%)
No	617 (88.4%)	356 (93.0%)	973 (90.0%)
**Wheezing categories**			
Never wheezing	341 (48.9%)	266 (69.5%)	607 (56.2%)
Early wheezing	188 (26.9%)	48 (12.5%)	236 (21.8%)
Late wheezing	82 (11.7%)	29 (7.6%)	111 (10.3%)
Persistent wheezing	69 (9.9%)	10 (2.6%)	79 (7.3%)
Missing	18 (2.6%)	30 (7.8%)	48 (4.4%)
**Asthma with recent exacerbation**			
Yes	47 (6.7%)	7 (1.8%)	54 (5.0%)
No	651 (93.3%)	376 (98.2%)	1027 (95.0%)
**Asthma with atopic disease**			
Yes	57 (8.2%)	16 (4.2%)	73 (6.8%)
No	641 (91.8%)	367 (95.8%)	1008 (93.2%)

**Table 3.** Associations between OH-PAH mixtures and asthma at age 8-9 years determined by logistic weighted quantile sum (WQS) regression models in which the direction of association is constrained to be positive (suggestive of adverse effects) or negative (suggestive of protective effects). The OH-PAH weights describe the proportional contribution of each metabolite to the WQS Index.

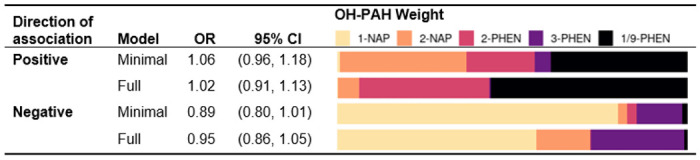

Note: Minimally adjusted models were adjusted for child age at assessment, child sex, study site, and urinary specific gravity. Fully adjusted models were additionally adjusted additionally adjusted for the interaction between SG and cohort, maternal age, education at enrollment, race, and ethnicity; season of birth; inflation-adjusted household income interacted with household size, neighborhood deprivation index, prenatal ETS exposure, postnatal ETS exposure, maternal history of asthma, and firstborn status.

## Data Availability

The data used for this study are not publicly available, but deidentified data may be available on request, subject to approval by the internal review board and under a formal data use agreement. Contact the corresponding author for more information. The computing code in R can be obtained from the corresponding author via email request.
